# A Container-Attachable Inertial Sensor for Real-Time Hydration Tracking

**DOI:** 10.3390/s19184008

**Published:** 2019-09-17

**Authors:** Henry Griffith, Yan Shi, Subir Biswas

**Affiliations:** Department of Electrical and Computer Engineering, Michigan State University, East Lansing, MI 48824, USA; shiyan3@msu.edu (Y.S.); sbiswas@egr.msu.edu (S.B.)

**Keywords:** non-wearable health monitoring sensors, automatic fluid intake monitoring, inertial sensors

## Abstract

Various sensors have been proposed to address the negative health ramifications of inadequate fluid consumption. Amongst these solutions, motion-based sensors estimate fluid intake using the characteristics of drinking kinematics. This sensing approach is complicated due to the mutual influence of both the drink volume and the current fill level on the resulting motion pattern, along with differences in biomechanics across individuals. While motion-based strategies are a promising approach due to the proliferation of inertial sensors, previous studies have been characterized by limited accuracy and substantial variability in performance across subjects. This research seeks to address these limitations for a container-attachable triaxial accelerometer sensor. Drink volume is computed using support vector machine regression models with hand-engineered features describing the container’s estimated inclination. Results are presented for a large-scale data collection consisting of 1908 drinks consumed from a refillable bottle by 84 individuals. Per-drink mean absolute percentage error is reduced by 11.05% versus previous state-of-the-art results for a single wrist-wearable inertial measurement unit (IMU) sensor assessed using a similar experimental protocol. Estimates of aggregate consumption are also improved versus previously reported results for an attachable sensor architecture. An alternative tracking approach using the fill level from which a drink is consumed is also explored herein. Fill level regression models are shown to exhibit improved accuracy and reduced inter-subject variability versus volume estimators. A technique for segmenting the entire drink motion sequence into transport and sip phases is also assessed, along with a multi-target framework for addressing the known interdependence of volume and fill level on the resulting drink motion signature.

## 1. Introduction

The underconsumption of water is a considerable global health concern [1,2]. Estimates suggest that 20% of adults exhibit significant dehydration, which is associated with numerous adverse health outcomes [3]. Recent evidence suggests that even mild underconsumption of water may have various negative health ramifications, including reduced cognitive function, obesity, and cancer [4]. The lack of an appropriate fluid intake is especially concerning for elderly individuals, due to the degradation of regulatory mechanisms with age [5].

To promote hydration maintenance, numerous sensing technologies have been demonstrated for tracking fluid consumption. Solutions include augmented drinking containers [6], which are currently available in the commercial marketplace, along with wearable [7] and contactless architectures [8]. Amongst these approaches, inertial measurement unit (IMU) sensors are a commonly employed sensing modality. IMU sensors have been used to implement motion-based consumption estimation strategies for both augmented container [9] and wearable [10] hydration trackers. 

Previous research has also proposed a container-attachable IMU sensor for hydration tracking applications [11]. This approach alleviates the restrictiveness of augmented containers by allowing for simplistic reconfiguration across multiple drinking vessels [12]. In addition, the non-wearable form factor of this device is especially promising for deployment amongst the at-risk elderly population, who may reject wearable solutions due to either preferences or various physical limitations [13]. The detection of drinking events for container-attached devices is also simplified versus wearable sensors, which may exhibit false alarms for arm movements exhibiting similar kinematics to drinking (i.e., eating, etc.) [14]. 

While IMU-based sensing is a promising solution for hydration tracking, prior work employing this modality has achieved only limited accuracy [14]. Moreover, while previous container-attachable studies have qualitatively described the relationship between the motion pattern of the bottle and drink volume [11], a direct description of the container’s inclination during drinking has not been used. Finally, while approaches estimating the fill level of a drink have been explored for alternative sensing architectures as a potential mechanism to improve the system’s performance, this approach has not been considered for an attachable sensing form factor [7]. 

**Figure 1:**
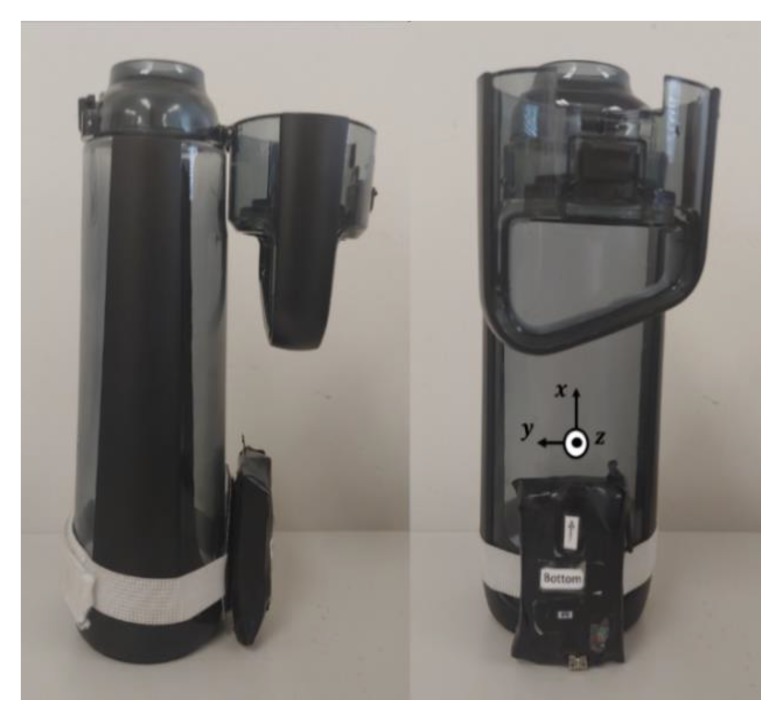
Refillable bottle with attached sensor prototype.

The research described herein seeks to address these limitations for an attachable triaxial accelerometer sensor. An image of the sensor prototype attached to the bottle used in the collections described within this manuscript is shown in Figure 1. 

By utilizing a feature space which more thoroughly describes the kinematics of the container during drinking, we seek to improve accuracy and reduce inter-subject variability versus prior studies utilizing only a limited motion description [14]. In addition, we seek to improve performance by forming aggregate consumption estimates based upon fill level estimates. As prior studies have demonstrated the ability to accurately classify fill levels with low resolution [3], we hypothesize that aggregate consumption estimates utilizing this quantity may improve performance. 

## 2. Related Work

Successful estimation of the fluid intake associated with a drinking event may be conceptualized as a two-stage process. Namely, the drinking event must first be segmented from the streaming sensor output (i.e., sip detection), followed by the estimation of drink volume from the partitioned data. While the prior task, which constitutes a particular case of online gesture detection or spotting [15], remains an area of active research interest (i.e., [10,16]), this review focuses solely on the latter portion of the problem statement. Details regarding drink event spotting for our container-attachable sensor can be found in [17]. 

Amft et al. [7] classified the initial fill level from which a drink was consumed using a magnetic coupling sensor system attached at both the shoulder and wrist. An experiment was conducted, in which three participants consumed 30 drinks from nine different container types in a scripted sequence. Drinks were consumed at three initial fill levels (full, half-full, and near empty), with subjects instructed to ingest only a minimal amount during each drink to avoid overconsumption. Individual-specific classifiers achieved an average classification accuracy of 72% across all subjects and container types. The classification accuracy varied considerably across subjects, ranging from 58% to 83%. While the estimation of aggregate consumption using fill level information is feasible, practical deployment requires increased resolution, along with a consideration of the effect of a varying drink volume on the estimation process. Moreover, the requirement of individual-specific training data limits the practical feasibility of this technique.

Mirtchouk et al. [18] estimated drink volume using multiple wearable audio and motion sensors. Sensors included an acoustic earbud, along with two commercial smartwatches and a commercial headset with embedded IMU sensors. Six participants consumed 171 drinks of multiple liquid types (i.e., coconut water, coffee, etc.) over a 72 hour period in an unscripted experiment intended to mimic real-world deployment. Regression models were trained using a leave-one-drink-out approach to account for the lack of consistent consumption patterns across participants. The mass of each drink was estimated with a best-case mean absolute percentage error (MAPE) of 47.2% under the assumption of a known fluid type. While multi-sensor systems are useful for laboratory collections, their feasibility for practical deployment is limited. 

To eliminate the multiple sensor requirement of prior work, Hamatani et al. [14] proposed FluidMeter, a tracking system utilizing the embedded IMU sensor within a commercial smartwatch. While the results for multiple experiments were reported, the Lab-micro+ dataset most closely resembles the experiments conducted herein. In this experiment, 16 individuals consumed 1069 drinks in a laboratory setting. The ground-truth mass of each drink was recorded using a digital scale. Linear regression models utilizing both the sip duration and the integral of the accelerometer signals not parallel to the forearm were employed to estimate drink mass. A best-case MAPE of 58.9% was achieved for the integration model trained using leave-one-subject-out (LOSO) validation. While variability across subjects was not reported for the Lab-micro+ dataset, models trained with this data exhibited considerable dispersion in accuracy across subjects (MAPE ranging from 57.9% to 11.0%) when applied to dedicated in-situ collection (Wild-office dataset). MAPE for the in-situ collection using ground-truth data collected with a commercial smart bottle was 31.8%. While Fluidmeter offers an unobtrusive mechanism for consumption tracking for existing smartwatch users, some individuals may refuse to adopt the requisite technology to employ this approach. Moreover, while the authors noted the influence of both fill level and drink volume on the resulting motion signature, no methods were explored to address this interdependence within the estimation process. 

Limited volume estimation results have been previously reported for the attachable architecture considered herein. Dong et al. [11] introduced the proposed sensing methodology, providing results for an experiment where seven subjects took 10 drinks from a refillable bottle. Various regression models using four hand-engineered features extracted from the accelerometer component parallel to the vertical axis of the bottle were evaluated. A best-case average aggregate consumption estimation error of 25% across subjects was achieved using support vector machine (SVM) regression models trained in a LOSO framework. A technique for estimating the relative drink mass using features describing the container’s inclination during drinking, denoted as the inclination signature (IS) feature set, was introduced in [19]. The utility of the proposed feature set for predicting the volume of each drink was evaluated by training an SVM classifier for a subset of the data described herein (1200 drinks) using a 90%/10% train/test split. For preliminary proof-of-concept, drink instances were classified according to their relative volume using various percentile-based partitions. For a median partition, a minimum classification error of 29.2% was achieved using a subset of the aforementioned feature set selected using filter-based techniques. 

Characteristics of the aforementioned literature are summarized in Table 1.

## 3. Materials and Methods

### 3.1. Data Collection

Eighty-four college-aged subjects (52 male, 32 female, aged 18–37) completed 161 trials of an experiment requiring the consumption of 12 drinks from a refillable 750 mL bottle shown in Figure 1. Subjects self-identified as healthy during the consent process. The proctor did not observe any motor deficiencies (i.e., tremors, etc.) influencing the drink motion pattern during any of the trials. Subjects were permitted to complete a maximum of four trials over multiple sessions. 

To begin the experiment, the bottle was filled to a consistent level, as determined visually by the experimental proctor. To ensure that a variety of drink volumes were captured, subjects were instructed to consume either a small, medium, or large drink prior to each sip, according to their personal preferences. The bottle was placed on an electronic scale following each drink to record the ground-truth mass. Variations from protocol (i.e., grasping and transporting the bottle without completing a drink, etc.) were noted by the proctor to allow for removal in pre-processing. The ground-truth fill level was estimated offline using knowledge of the bottle’s geometry. Two trials were discarded after collection due to hardware failure, yielding a total valid data set of 159 trials (1908 drinks). 

**Figure 2:**
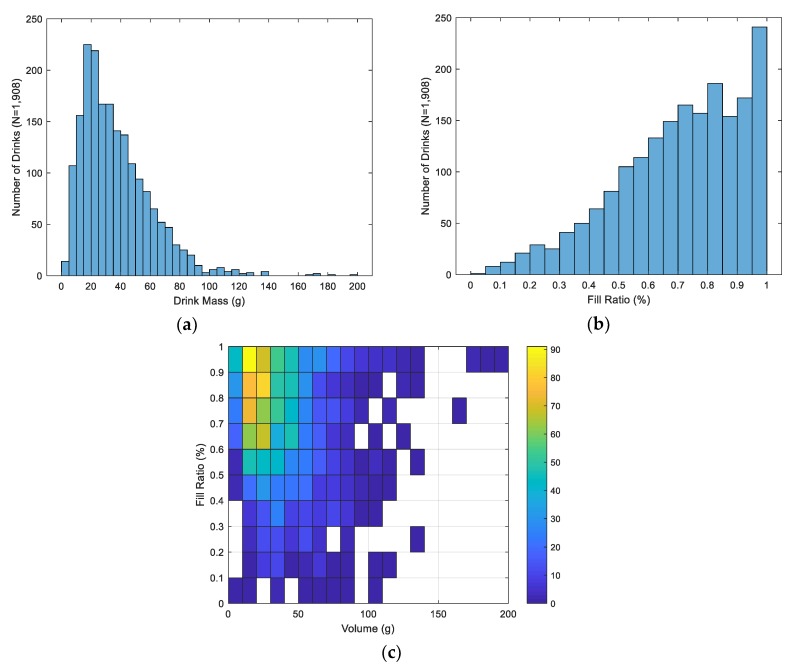
(**a**) Univariate distribution of drink masses; (**b**) univariate distribution of fill ratios; (**c**) joint distribution of masses and fill levels.

All subject recruitment, data collection, and record storage was conducted according to protocol approved by the Institutional Research Board at Michigan State University. The univariate distribution of the initial fill ratio (fill level normalized to total fillable height) and mass of each drink collected, along with their joint distribution, are depicted in Figure 2. As shown, drink instances are skewed towards larger fill ratios due to the protocol of filling the bottle completely at the beginning of each trial. The volume distribution is right skewed due to the variability in large drink consumption across individuals.

### 3.2. Hardware

A prototype sensor module consisting of a triaxial accelerometer (ADXL345, Analog Devices, Norwood, MA, USA), gyroscope (InvenSense IMU-3000, TDK, San Jose, CA, USA), and 802.15.4 wireless transceiver (IRIS Mote Module, MEMSIC, Andover, MA, USA) was connected to the refillable bottle, as depicted in Figure 1, for all experiments. Only data from the accelerometer was used within the current work. The sensor was placed beneath the lid at the bottom of the bottle to avoid interference with gripping. The local coordinate frame of the sensor was aligned with the bottle geometry as follows: (1) the *x* axis was aligned parallel to the vertical axis of the bottle, such that the accelerometer signal only consisted of the static acceleration due to gravity when the bottle was placed stationary on a level surface (i.e.: **a** = g**x**), (2) the *y* and *z* axes were aligned parallel and normal to the surface of the bottle, respectively.

**Figure 3:**
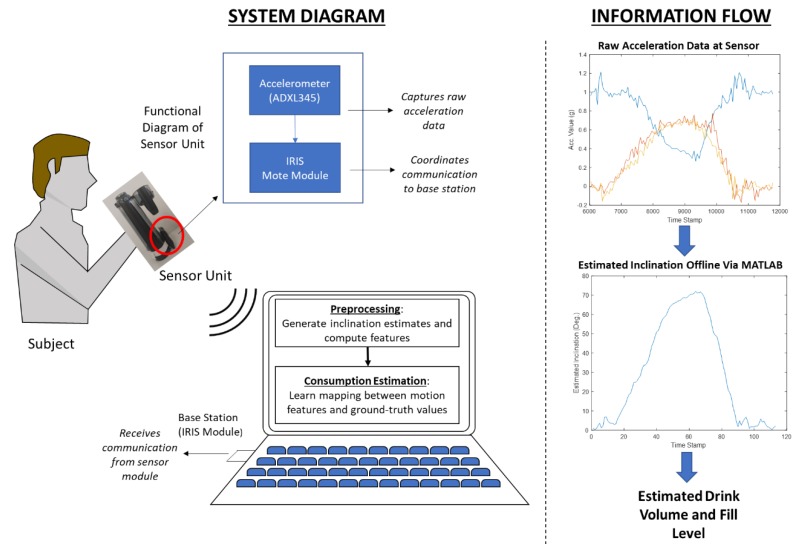
System and information flow diagram.

Data from the sensor module was transmitted to a base station (IRIS Wireless measurement system, MEMSIC, Andover, MA, USA) connected to a PC via USB at a target polling frequency of 20 Hz. All files were processed offline using MATLAB (Mathworks, Natick, MA, USA). A diagram of the collection system is shown in Figure 3.

### 3.3. Preprocessing

To begin preprocessing, the bias of each component was estimated by averaging the initial 50 samples of each recording while the bottle was stationary. Portions of the signal corresponding to variations in protocol were then removed. Each file was subsequently parsed into macro-events using a threshold-based algorithm exploiting the stationary placement of the bottle between drinks. This process captured the entire interval for which the bottle was in motion (i.e., both transport to and from the mouth, along with sipping). 

**Figure 4:**
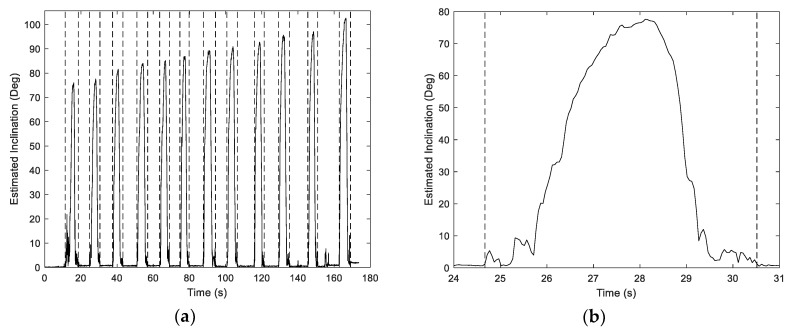
(**a**) Variation in estimated container inclination over an experimental trial (wide view); (**b**) zoom view for drink 2.

After partitioning, signals were resampled to the target frequency of 20 Hz to account for variability in the base station polling interval. Smoothing was performed using a two-sample moving average filter to mimic the frequency response of the original work conducted in [11]. The sensor’s inclination with respect to gravity, which is equivalent to that of the container under ideal sensor alignment, was then estimated under the assumption of negligible dynamic acceleration, as specified in (1):

(1) $$\theta =\tan^{-1}\frac{\sqrt{a_{y}^{2}+a_{z}^{2}}}{a_{x}}$$

where $a_{j}$ denotes the conditioned *j*th component of the accelerometer. Variation in the inclination over a single trial is depicted in Figure 4. 

### 3.4. Micro-Event Partitioning 

Further partitioning was conducted to isolate the drinking portion of the total segmented event. As described in similar work (i.e., [14]), this segmentation was motivated by the substantial variation that may occur in the transport motion pattern, depending on the specific drinking scenario, which does not necessarily influence drink volume. For the experiments described herein, variability in the transport motion pattern could be associated with the order of the drink within the trial (i.e., more careful handling for full containers, more rapid transport as the subject becomes familiar with protocol, etc.). In addition, differing orientation of the container upon retrieval may also introduce variability. 

To isolate the drinking portion of the event, the asymmetry of the container about its vertical axis was exploited. Namely, as the lid of the container encourages consumption from the opposite edge, we hypothesized that while axial rotations may occur during transport, such rotations would be minimized during drinking to avoid spilling. As originally demonstrated in [20], these rotations may be quantified by computing the sensor’s angular position within the cross-sectional plane of the bottle, as specified in (2).

(2) $$\alpha =\tan^{-1}\left(\frac{a_{z}}{a_{y}}\right)$$

**Figure 5:**
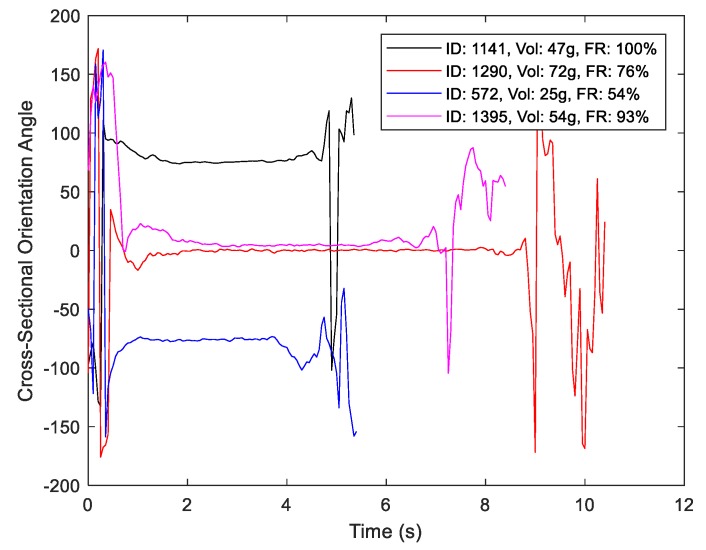
Position of the sensor in the cross-sectional plane of the bottle for four randomly chosen drinks (stationary intervals indicate a lack of rotation about the vertical axis of the bottle).

As shown for a random sample of drinks in Figure 5, α maintains a relatively constant value near the middle of each event. This stationary interval is used to define the following micro-event partition:

- *Lift*: The portion of the macro-event proceeding the sip micro-event;
- *Sip*: The portion of the micro-event for which α is estimated as stationary;
- *Place*: The remainder of the macro-event after termination of the sip micro-event.

**Figure 6:**
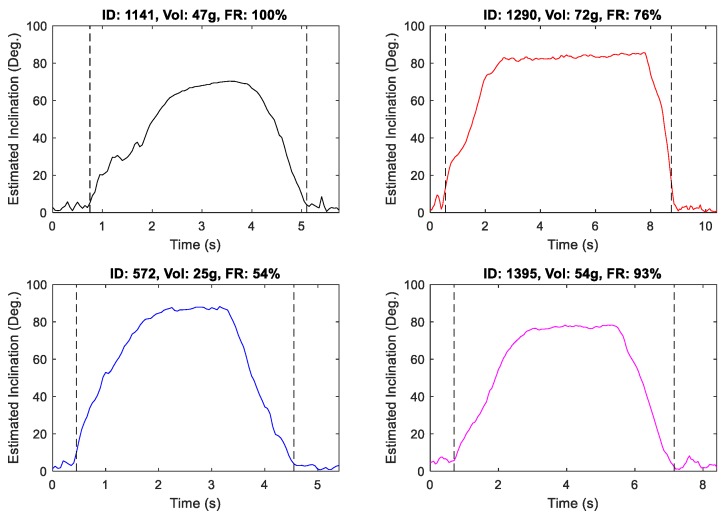
Micro-event partitions for Figure 5 drinks (dashed vertical lines indicate boundaries of sip micro-event).

To isolate the sip micro-event, the sample-over-sample difference signal of α was initially thresholded to a maximum value of 8 degrees. All intervals meeting the threshold criteria which were separated by less than two samples were then merged, with the largest resulting interval extracted. The resulting micro-partition for the four random drink events depicted in Figure 5 is shown in Figure 6. 

### 3.5. Feature Engineering 

A set of 33 hand-engineered features describing the container’s inclination during drinking were developed based upon visual observations during the experiment and resulting inclination estimates. In addition to key kinematic quantities (i.e., maximum inclination, maximum rate of inclination, etc.), amplitude values of both the raw and normalized curves were binned to create a low-level time-invariant description of the signal. Previously denoted as the IS feature set [11], the version employed herein has been slightly modified from its original form to employ exclusive binning in order to reduce the correlation between features. This modified feature set is summarized in Table A1 (Appendix A). 

For purposes of benchmarking, linear regression (LR) models utilizing previously identified characteristic motion features (i.e., sip duration and integral of inclination) [14] were also evaluated. While motivated by the methods of [14], it should be re-emphasized that a direct comparison is not applicable due to differences in the sensor placement (i.e., wearable versus attachable). 

### 3.6. Machine Learning

To promote comparability with prior work, leave-one-trial-out (LOTO) validation was performed. This approach is consistent with the target use case, where models trained on a broad pool of users would be employed on a new user absent of customization. While a LOTO approach includes some subject-specific training data, the magnitude of this contribution is limited (i.e., maximum subject-specific training data of 1.9%). LOTO was chosen versus a LOSO approach to ensure that the sizes of the training and testing sets were consistent across evaluations. 

A set of support vector machine (SVM) regression models was trained for both volume and fill ratio estimation. Linear, medium (kernel scale = 5.7), and coarse (kernel scale = 23) Gaussian kernel functions were considered. Hyperparameters were set to the default values, as established in MATLAB’s Regression Learner toolbox. SVMs were chosen based upon their superior performance for the current sensor architecture in [11], where they outperformed both neural network and tree-based regression models. Features were standardized using z-score normalization.

### 3.7. Performance Metrics 

Multiple performance metrics were used to assess the quality of the estimation on both a per-drink and aggregate basis. MAPE was used to quantify the estimation performance on a per-drink basis. This metric was chosen over alternative measures (i.e., root mean squared error, etc.) due to its utilization in prior work (i.e., [14,18]). For assessing the aggregate (i.e., multi-drink) consumption estimation accuracy, mean overall absolute percentage error (MOAPE) was used. Within the remainder of this manuscript, MOAPE(*n*) denotes the computation of MOAPE after the completion of *n* drinks within a trial. While MAPE provides the most rigorous assessment of the model’s performance, MOAPE is useful for exploring utility in practical scenarios where aggregate consumption is of primary concern (i.e., estimating total daily consumption, estimating consumption during exercise, etc.).

## 4. Results and Discussion

### 4.1. Micro-Event Partitioning Analysis

The correlation between micro-event durations and drink volume is provided in Table 2. Consistent with the hypothesis presented in Section 3.4, sip duration is more strongly correlated with volume versus the two transport durations. Correlations between sip duration, along with the previously proposed motion feature related to the integral of the inclination [14], are shown in Table 3 for various ranges of controlled fill levels.

Results are consistent with those presented in [14], which reported a correlation coefficient with drink volume of 0.69 and −0.60/−0.55 for sip duration and the integral of accelerometer signals not parallel to the forearm (which were denoted as being related to container inclination), respectively. Moreover, it should be noted that the strength of the correlation between both motion features and volume increases when the fill level is restricted within a narrower range of values. This result is also consistent with prior work [14], which denoted an influence of both volume and fill level on the resulting motion signature. The utility of the proposed micro-event partitioning process for volume estimation is explored in the remainder of this section. 

### 4.2. Volume Estimation

**Figure 7:**
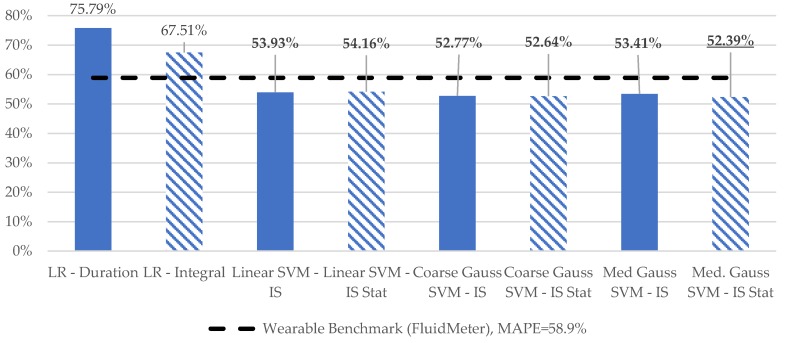
Variation in volume mean absolute percentage error for various models considered.

Variation in MAPE for the various learning models considered is depicted in Figure 7. Models computed for the sip micro-event only are labeled as Stat., with all other reported results computed for the entire macro-event duration. Consistent with the wearable results in [14], LR models employing the integral of inclination outperformed those using duration. The level of improvement is enhanced versus the results presented in [14]. We hypothesize that this difference is associated with the use of the inclination estimate of the container, as opposed to the individual accelerometer channels which are related to this quantity. 

All SVM models outperformed the simplistic single factor LR motion models. Moreover, all SVM models exhibited superior performance to the previous best-case reported MAPE of 58.9% for a single wearable sensor in an experiment using scale-based ground-truth [14]. Only minimal differences in MAPE were observed for models utilizing the proposed sip micro-event segmentation versus those computed based on the entire drinking event.

**Figure 8:**
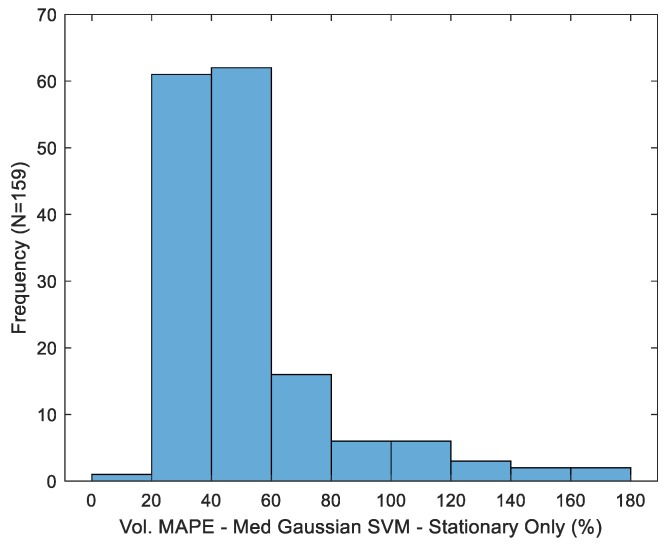
Distribution of volume mean absolute percentage error across trials for the best-case estimator.

Variation in MAPE across trials is depicted in Figure 8 for the best-case volume estimator (medium kernel, sip micro-event partition). Consistent with prior observations [14], dispersion in the observed error metric is substantial, with a standard deviation of 28.18%. 

Volume MOAPE for varying drink sequence lengths is presented in Table 4. The aggregate estimation accuracy generally improves with an increased sequence length, with reductions being more pronounced for the proposed IS-based SVM models. While not directly comparable due to the employment of the more stringent MOAPE cumulative metric herein, the best-case aggregate consumption estimation accuracy of 19.49% is improved versus the average value of 25% reported in [11]. 

**Figure 9:**
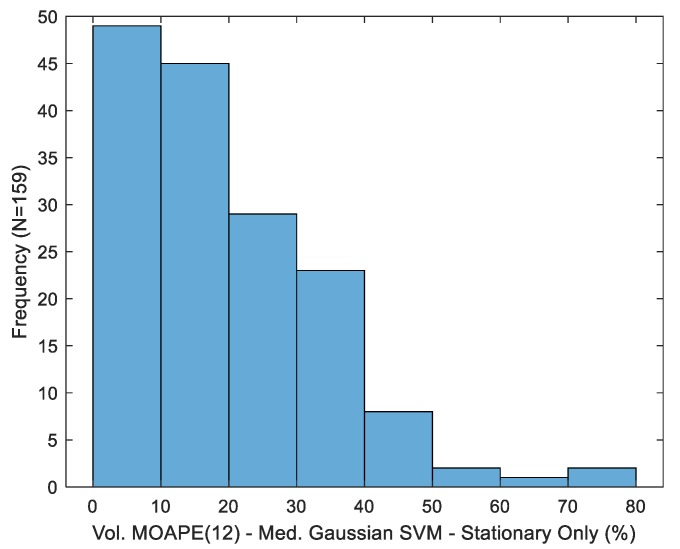
Distribution of volume mean overall absolute percentage error after 12 drinks for the best-case estimator.

While the best-case MOAPE(12) value exceeds that computed for the in-the-wild data set reported in [14] (16.95%), direct comparability is limited by the inclusion of potential sip detection-related errors (i.e., both false alarms and missed drink detections) in this latter metric, along with the utilization of a commercial smart-bottle for ground-truth labeling (the vendor only reports the accuracy of “a fraction of an ounce” for this device). Variability of the best-case aggregate estimator (medium kernel, entire macro-event duration) is presented in Figure 9. Similar to MAPE, inter-subject variability is considerable (standard deviation of 14.75%). For purposes of comparison, the standard deviation across participants for the in-the-wild dataset in [14] was 14.17%.

### 4.3. Fill Level Estimation

**Figure 10:**
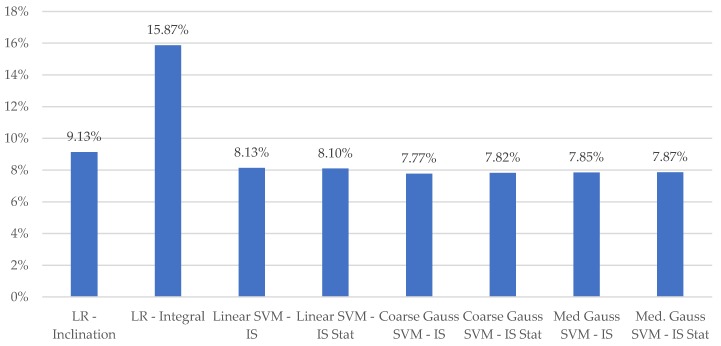
Variation in the fill ratio mean absolute percentage error for the various models considered.

**Figure 11:**
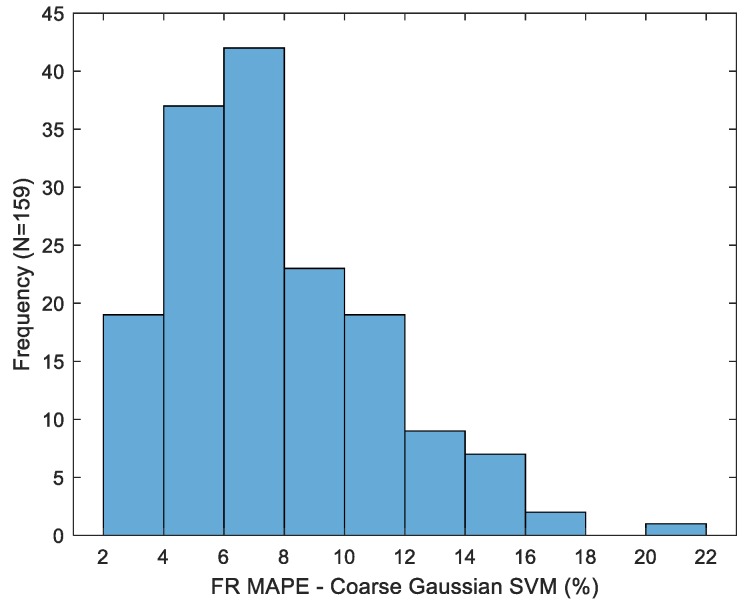
Distribution of the fill ratio mean absolute percentage error across trials for the best-case estimator.

Variation in fill ratio MAPE for the multiple models considered is depicted in Figure 10. Sip duration was replaced with maximum inclination for a single-factor LR benchmark model due to its strong correlation with the fill ratio. This relationship is emphasized by the considerable increase in this quantity over the course of the experiment, as shown in Figure 4. Fill ratio estimation accuracy is greatly improved versus volume prediction for both the single factor regression and more complex SVM models. Variability in MAPE for the best-case estimator (coarse kernel, entire macro-event partition) is shown in Figure 11. Error dispersion across trials is greatly reduced versus volume estimators. Namely, the fill ratio MAPE standard deviation is 3.39%, versus 28.18% for the best-case volume MAPE.

**Figure 12:**
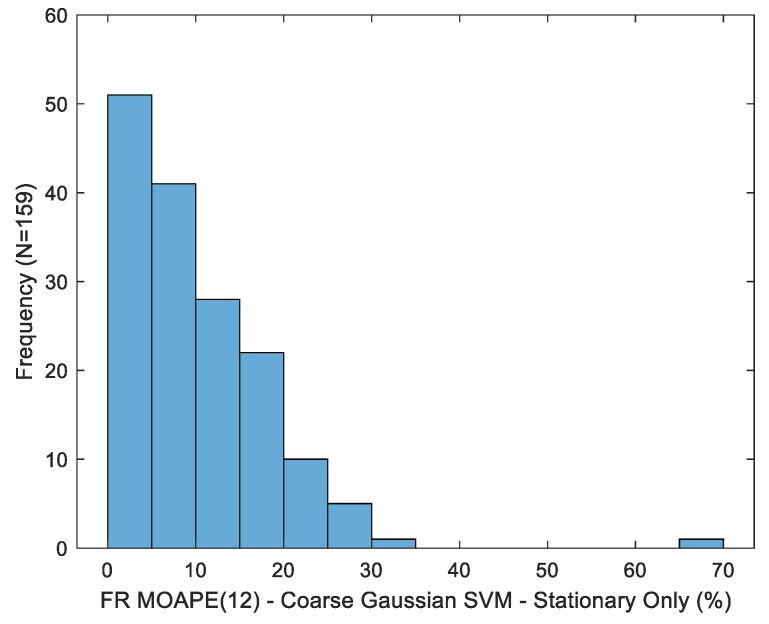
Distribution of the fill ratio mean overall absolute percentage error after 12 drinks for the best-case estimator.

The fill ratio MOAPE is shown in Table 5 for varying drink sequence lengths. In contrast to volume estimation, the point nature of fill ratio estimates does not allow for sequential error cancelation across multiple drinks. Accuracy generally degrades with an increasing sequence length. We hypothesize that this discrepancy might be associated with the aforementioned skewing of training data towards larger fill ratios. The variability in fill ratio MOAPE(12) estimates across trials is depicted in Figure 12 for the best-case estimator (coarse kernel, sip micro-event), with a standard deviation of 8.58% observed (versus 14.75% for volume MOAPE(12) estimates).

### 4.4. Residual Volume Estimation

Fill ratio estimates for pairs of drinks may be used to estimate the aggregate inter-drink consumption for a known container geometry, as specified in (3), where ${\hat{V}}_{i:f}$ denotes the estimated aggregate consumption from drink i to f; β is a container-specific linear density parameter; and $v_{k}$ and ${\hat{FR}}_{k}$ denote the ground-truth volume and estimated fill ratio at the initiation of drink k, respectively. 

(3) $${\hat{V}}_{i:f}\approx \overset{f}{\underset{k=i}{\sum }}v_{k}\approx \beta \left({\hat{FR}}_{i}-{\hat{FR}}_{f+1}\right)$$

**Figure 13:**
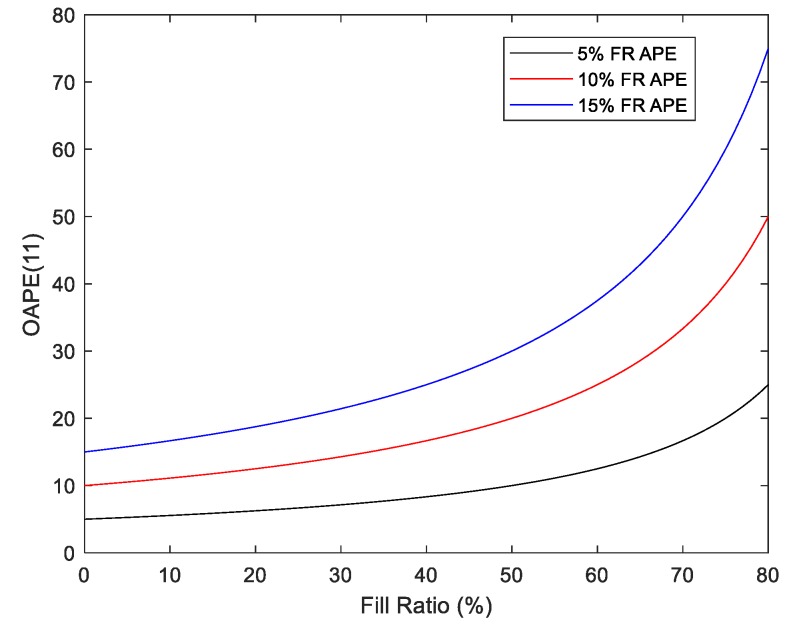
Residual volume-based overall average percentage error versus final fill ratio for various fill ratio absolute percentage error values.

This mechanism, hereby denoted as residual volume estimation, was assessed herein based upon the noted superior accuracy and reduced inter-subject variability of fill ratio versus volume estimators. The comparison was performed using the MOAPE(11) metric. This sequence length was chosen as it represents the maximum number of drinks which can be assessed using initial fill ratio estimates for our 12-drink protocol. As shown in Table 6, the enhanced accuracy of fill ratio estimates does not improve aggregate consumption estimates versus those formed through the summation of drink-level volume estimates (hereby denoted as cumulative consumption estimation). This discrepancy is attributed to the ability of the latter method to benefit from the cancelation of sequential estimation errors within a drink sequence. Moreover, normalization effects during the conversion to the aggregate consumption volume (i.e., residual volume-based OAPE) serve to distort the achieved accuracy in fill ratio estimation (i.e., fill ratio APE). This distortion is more pronounced for trials with smaller levels of aggregate consumption, as emphasized in Figure 13 and specified in (4).

(4) $$OAPE\left(j\right)=\frac{\left|{\hat{FR}}_{j+1}-FR_{j+1}\right|}{1-FR_{j+1}}=\frac{APE_{j+1}}{1-FR_{j+1}}$$

### 4.5. Multi-Target Estimation Framework

Based upon the observations summarized in Table 3, various techniques were explored for developing volume estimators incorporating fill ratio information. The first approach conditioned the training set using the estimated fill ratio. Namely, training data was restricted to the 150 samples whose fill level labels were closest to the estimated fill ratio in the Euclidean sense. While the computational overhead of this approach is not feasible in practical deployment, similar techniques could be realized by selecting from a pretrained model library for targeted fill ratio ranges. 

To explore the maximum achievable benefit of this approach, an analysis was conducted using ground-truth fill ratio information in addition to estimates. Moreover, to assess the utility of explicitly mandating this form of fill ratio incorporation, a strategy of appending the fill ratio into the feature space was also considered. The results for all four analysis combinations are presented in Table 7 for the best-case macro-event volume estimator (coarse Gaussian SVM). Estimated fill ratios were obtained using the coarse Gaussian SVM regression model. As demonstrated, while ground-truth fill ratio information improves volume estimation accuracy, no benefit is realized using noisy fill ratio estimates. Moreover, the proposed approach of restricting training data to examples with similar fill ratios produced only minimal error reduction versus feature space expansion. We hypothesize that this limitation is associated with the reduction in available training data using the prior method. 

### 4.6. Limitations

Although the proposed attachable architecture offers notable advantages versus competing approaches, it is characterized by some fundamental limitations. Namely, the sensing approach described herein is restricted to drinking vessels in which flow is introduced through inclination (i.e., no straw-based containers, etc.). Moreover, the attachable device limits ubiquity versus wearable sensors, due to the requirement that dedicated hardware be manually repositioned on the container before each drinking episode. 

Beyond these innate restrictions, generalization of the results presented herein is limited by the scripted nature of the experiment. Further analysis exploring the efficacy of the proposed approaches for the intended use case, which includes the requirement of drink spotting amongst potentially confounding activities (i.e., handling, bottle maintenance, etc.), is required. Moreover, the described techniques should also be assessed for more natural (i.e., no scripted volume prompts) consumption patterns. Finally, experiments should be conducted for additional types of drinking containers (i.e, mugs, glasses, etc.). 

### 4.7. Future Work

Future work will focus on further analysis of the collected dataset, including the incorporation of gyroscope data within the estimation process. In addition, an analysis of the effect of sensor placement on the estimation performance using a subset of data for which two sensors were attached at varying positions will be conducted. Furthermore, more sophisticated analysis techniques, including the application of end-to-end learning strategies, along with more complete multi-target regression approaches yielding the joint estimation of both volume and fill ratio [21] will be explored. In addition to analyzing existing data, further data collection addressing the mentioned limitations in the prior subsection will be conducted. 

## 5. Summary 

A container-attachable IMU sensor for tracking fluid consumption was demonstrated herein. Consumption estimates were formulated using support vector machine regression models with hand-engineered features describing the inclination of the container during drinking. Results were presented for an experiment consisting of 1908 drinks consumed by 84 participants. MAPE was reduced by 11.05% on a per-drink basis versus the prior state-of-the-art for a single wearable IMU in an experiment utilizing scale-based ground-truth values [14]. A MOAPE of 19.49% was achieved over the 159 trials conducted. This multi-drink estimation accuracy improves upon previously reported results for a container-attachable sensing architecture [11].

Consistent with prior motion-based studies, errors in volume estimates were shown to demonstrate considerable inter-subject variability, as quantified by a MAPE standard deviation of 28.18% for the best-case estimator. As a possible alternative tracking approach, aggregate consumption estimates using fill level information were explored. Fill ratio estimates were shown to exhibit an improved accuracy (best-case MAPE of 7.77%) and reduced inter-subject variability (corresponding standard deviation of 3.39%) versus volume estimates. Aggregate consumption estimates based upon the fill ratio did not exhibit an improved accuracy versus those obtained through the summation of individual drink volume estimates.

In addition, a technique for segmenting the entire drink motion sequence into transport and sip micro-events was proposed and demonstrated. While this micro-partitioning did not considerably affect the estimation accuracy for the scripted results considered herein, it may be useful for in-the-wild applications where variability in transport motion patterns is enhanced. Furthermore, the possibility to improve volume estimation accuracy by exploiting the influence of fill level on the resulting motion pattern was investigated. While the inclusion of ground-truth fill ratio data as both an additional feature and conditioning factor in training data was shown to improve the accuracy, the utilization of noisy fill ratio estimates provided no performance improvement.

## Figures and Tables

**Table 1:** Summary of related work reporting volume estimation results on a per-drink basis.

Paper Identifier	Sensing Modality	Estimation Quantity/Approach	# of Subjects/ # of Drinks	User-Specific vs. Out-of-Subject Model	Best Reported Per-Drink Performance Metric
Amftet et al. [7]	Wearable magnetic coupling sensors on wrist and shoulder	Fill level classification (3 levels: full, medium, near empty)	3 subjects/ 810 drinks	User-Specific	72% classification accuracy
Mirtchouket et al. [18]	Earbud, two smart watches, smart glasses with embedded IMUs	Volume regression	6 subjects/ 285 drinks	Mixed (i.e., both user-specific and out-of-subject training data)	47.2% MAPE
Hamataniet et al. [14]: Lab-micro+ collection	Commercial smartwatch with embedded IMUs	Volume regression	16 subjects/ 1069 drinks	Out-of-subject (user-specific results reported for benchmarking)	58.9% MAPE
Hamataniet et al. [14]: Wild office dataset	Commercial smartwatch with embedded IMUs	Volume regression	16 subjects/ 178 drinks	Out-of-subject, with models trained on Lab-micro + data and ground-truth collected via commercial smart bottle	34.6% MAPE
Griffith et al. [19]:	Bottle-attachable IMU Sensor	Binary volume classification with median volume partition	64 subjects/ 1200 drinks	Mixed (i.e., both user-specific and out-of-subject training data)	29.2% classification error for median partition
Current Manuscript	Bottle-attachable IMU Sensor	Volume and fill level regression	84 subjects/ 1908 drinks	Out-of-subject	52.4% MAPE (volume regression)

**Table 2:** Correlation between features and volume label.

Micro-Event Duration	Pearson Correlation Coefficient (Corr. Coeff.) (Entire Dataset)
*Lift Duration*	0.189
*Sip Duration*	0.449
*Place Duration*	0.159

**Table 3:** Correlation between previously reported motion features and volume for various fill ratio (FR) ranges.

Motion Feature	Corr. Coeff. (Entire Dataset *N* = 1908)	Corr. Coeff. (FR > 50% *N* = 1576	Corr. Coeff. (FR > 70% *N* = 1075)	Corr. Coeff. (FR > 90% *N* = 413)
*Sip Duration*	0.449	0.457	0.471	0.557
*Integral of Inclination Over Sip Duration*	0.536	0.543	0.571	0.672

**Table 4:** Variation in volume mean overall absolute percentage error for multiple prompt periods (bold values emphasize the best performing model for each period).

Model Identifier	MOAPE(3)	MOAPE(6)	MOAPE(9)	MOAPE(12)
*Duration Only–LR*	36.74%	34.41%	33.51%	32.42%
*Integral Only–LR*	**28.68%**	27.76%	27.59%	27.79%
*IS–Linear SVM*	32.87%	26.40%	23.44%	21.46%
*IS Stat.–Linear SVM*	33.74%	26.64%	23.52%	21.56%
*IS–Coarse Gaussian SVM*	31.55%	25.49%	22.58%	20.75%
*IS Stat.–Coarse Gaussian SVM*	31.79%	25.39%	22.48%	20.65%
*IS–Medium Gaussian SVM*	30.52%	**24.98%**	**21.62%**	19.64%
*IS Stat.–Medium SVM*	30.55%	24.86%	21.71%	**19.49%**

**Table 5:** Variation in the mean overall absolute percentage error for multiple prompt periods—fill ratio estimation (bold values emphasize the best performing model for each period).

Model Identifier	MOAPE(3)	MOAPE(6)	MOAPE(9)	MOAPE(12)
*Max. Inclination Only–LR*	10.90%	**7.64%**	8.12%	12.57%
*Integral Only–LR*	18.20%	9.14%	13.27%	22.88%
*IS–Linear SVM*	8.82%	8.18%	7.99%	9.95%
*IS Stat.–Linear SVM*	9.29%	8.30%	8.20%	10.28%
*IS–Coarse Gaussian SVM*	8.82%	8.18%	7.99%	9.95%
*IS Stat–Coarse Gaussian SVM*	8.68%	7.99%	**7.96%**	**9.86%**
*IS–Medium Gaussian SVM*	8.24%	8.22%	8.04%	10.80%
*IS Stat.–Medium SVM*	**7.98%**	7.87%	8.08%	11.10%

**Table 6:** Comparative mean overall absolute percentage error after 11 drinks: residual versus cumulative volume estimation approach.

Model Identifier	Residual Volume Technique (FR-Based)	Cumulative Volume Technique (Volume-Based)
*Linear SVM—IS*	28.10%	21.65%
*Coarse Gauss SVM—IS*	26.84%	20.79%
*Med Gauss SVM—IS*	29.04%	20.01%

**Table 7:** Volume estimation accuracy enhancement using fill ratio information (baseline value with no fill ratio information: 52.77%).

Strategy for Incorporating Fill Ratio Information	Using Ground-Truth Fill Ratio Values	Using Estimated Fill Ratio Values
*Partition Training Set*	48.59%	54.99%
*Add as Feature*	49.90%	52.66%

## Appendix A

The inclination signature feature set is specified in Table A1, where $\theta^{j}=\left\{\theta_{1},\theta_{2},\;\ldots ,\theta_{D}\right\}$ denotes the inclination estimate of the *j*th drink event defined according to (1).

**Table A1:** Inclination signature (IS) feature set.

Feature ID	Feature Symbol	Feature Definition	Description
***1***	$\theta^{*}$	$max\left(\theta^{j}\right)$	Maximum inclination angle during drink event
***2***	***D***	***length***($\theta^{j}$)	Duration of drinking event
***3–11***	$A_{\theta_{k}}$	$count\left(\theta^{j}<T\left(k\right)\right),\;k=1$ $count\left(T\left(k-1\right)\leq \theta^{j}<T\left(k\right)\right)$ k∈{2,7} $count\left(\theta^{j}\geq T\left(k\right)\right),\;k=8$ $T\left(k\right)\in \left\{20^{{}^{\circ}},\;\ldots ,\;90^{{}^{\circ}}\right\}$	Number of samples for which inclination angle satisfies specified amplitude range criteria
***12–20***	$AR_{P_{k}}$	$count\left(\frac{\theta^{j}}{\theta^{*}}<P\left(k\right)\right),\;k=1$ $count\left(P\left(k-1\right)\leq \frac{\theta^{j}}{\theta^{*}}<P\left(k\right)\right),\;$ k∈{2,7} $count\left(\frac{\theta^{j}}{\theta^{*}}\geq P\left(k\right)\right),\;k=8$ P(k)∈{20%, …, 90%}	Number of samples for which normalized inclination angle satisfies relative amplitude criteria
***21***	$Q_{\theta }$	$\frac{\theta^{*}}{D}$	Ratio of maximum inclination value to duration
***22***	$\bar{\theta }$	$mean\left(\theta^{j}\right)$	Mean inclination angle
***23***	$D_{\theta_{R}}$	$\frac{argmax\left(\theta^{j}\right)}{D-argmax\left(\theta^{j}\right)}$	Ratio of time for which inclination angle is increasing relative to decreasing
***24–25***	$S_{\theta },\;S_{\theta }^{T}$	$T\sum_{m=1}^{U}\theta_{m}^{j}$	Riemann sum approximation to integral of inclination curve over entire duration (U=D) or inclination interval ($U=argmax\left(\theta^{j}\right)$)
***26***	$RE_{\theta }$	$\frac{\theta^{*}-\theta_{1}}{argmax\left(\theta^{j}\right)-1}$	Slope of line intersecting inclination trajectory start of trajectory time of maximum value
***27***	$FE_{\theta }$	$\frac{\theta_{D}-\theta^{*}}{D-argmax\left(\theta^{j}\right)}$	Slope of line intersecting inclination trajectory at time of maximum value and end of trajectory
***28/29***	${\theta_{T}^{\prime }}^{*}$/ ${\theta_{A}^{\prime }}^{*}$	$\max\left({\theta {\left(1:argmax\left(\theta^{j}\right)\right)}^{\prime }}^{j}\right)$/ $\max\left({\theta {\left(argmax\left(\theta^{j}\right):D\right)}^{\prime }}^{j}\right)$	Maximum rate of inclination/declination, where $\theta^{\prime j}$ is a numerical estimate of the derivative of $\theta^{j}$
***30/31***	${\bar{\theta }}_{T}^{\prime }$/ ${\bar{\theta }}_{A}^{\prime }$	$mean\left({\theta {\left(1:argmax\left(\theta^{j}\right)\right)}^{\prime }}^{j}\right)$/ $mean\left({\theta {\left(argmax\left(\theta^{j}\right):D\right)}^{\prime }}^{j}\right)$	Mean rate of inclination/declination
***32/33***	$s_{\theta_{T}^{\prime }}$/ $s_{\theta_{A}^{\prime }}$	$std\left({\theta {\left(1:argmax\left(\theta^{j}\right)\right)}^{\prime }}^{j}\right)$/ $std\left({\theta {\left(argmax\left(\theta^{j}\right):D\right)}^{\prime }}^{j}\right)$	Standard deviation of inclination/declination rate
